# T189. PEPTIDE SHARING BETWEEN SCHIZOPHRENIA-RELATED PROTEINS AND THE INFLUENZA A VIRUS MAY OFFER A WINDOW INTO THE IMMUNE AETIOLOGY OF PSYCHOTIC DISORDERS

**DOI:** 10.1093/schbul/sby016.465

**Published:** 2018-04-01

**Authors:** Adrianna Kepinska, Thomas Pollak, Conrad Iyegbe, Robin Murray

**Affiliations:** Institute of Psychiatry, Psychology & Neuroscience, King’s College London

## Abstract

**Background:**

Schizophrenia is a complex disorder in which infection and immune mechanisms are thought to play a role. Epidemiological and ecological studies have implicated influenza infection in particular and it is possible that cross-reactivity, or molecular mimicry, between the influenza virus and brain proteins underlies this association. Proteins might share amino acid sequences, which could thus provide the basis for an autoimmune response that targets endogenous proteins. This study is the first to characterise sequence alignment between schizophrenia-related brain proteins and the proteome of the influenza A virus, and comparing it with sequence alignment in proteins not implicated in schizophrenia.

**Methods:**

The software Peptide Match Service (https://research.bioinformatics.udel.edu/peptidematch/index.jsp; Protein Information Resource, University of Delaware and Georgetown University Medical Center) was used to obtain sequence alignments between protein sequences. A case-control study design was used to compare schizophrenia-related proteins to proteins not involved in schizophrenia. Schizophrenia-related proteins were operationalised as proteins found significant in the Psychiatric Genomics Consortium schizophrenia genome-wide association studies (GWAS). The control group consisted of null proteins (p-value > .75) in the GWAS. Null proteins were also selected to represent genes expressed in tissues other than central nervous system tissues. Both groups were equalised for the total amino acid count. Perfect pentapeptide matches (i.e. 5 amino acids) in proteins and the influenza proteome were explored.

**Results:**

There was a link between schizophrenia-related (GWAS-significant) proteins and presence of perfect matches between proteins and the influenza proteins polymerase acidic protein (χ2 (1) = 5.284, p = .022, two-sided) and RNA-directed RNA polymerase catalytic subunit (χ2 (1) = 6.132, p = .013, two-sided). Pentapeptide-sharing was found to be highly significant between schizophrenia-related proteins and the hemagglutinin precursor (χ2 (1) = 17.723, p = .000026, two-sided). There was no significant difference (p > .05) between schizophrenia-related proteins and proteins not implicated in schizophrenia (GWAS-null proteins) in the frequency of proteins having perfect matches with the influenza A proteins PB2-S1, polymerase basic protein 2, matrix protein 1 and 2, and neuraminidase. However, the result for matrix protein 1 approached statistical significance (χ2 (1) = 3.319, p = .068, two-sided).

**Discussion:**

We find evidence to suggest there is significant overlap between the linear structures of proteins involved in schizophrenia and those integral to the influenza virus. Future research should establish the biological relevance of this finding, particularly regarding the antigenicity of the peptide sequences which we have identified. Extra studies should also go beyond sequences and address structural homologies. Future research could assess whether an immune reaction against particular schizophrenia-related proteins is a plausible mechanism contributing to psychotic disorders. Also, exploring peptide sharing in different influenza strains could offer insights into links between influenza pandemics, maternal infection, and psychosis. Elucidating peptide sharing might have implications for schizophrenia risk management and safe influenza prevention.

